# Changing hearts and minds: theorizing how, when, and under what conditions three social influence implementation strategies work

**DOI:** 10.3389/frhs.2024.1443955

**Published:** 2024-09-05

**Authors:** Bryan J. Weiner, Rosemary D. Meza, Predrag Klasnja, Rebecca Lengnick-Hall, Gretchen J. Buchanan, Aaron R. Lyon, Kayne D. Mettert, Marcella H. Boynton, Byron J. Powell, Cara C. Lewis

**Affiliations:** ^1^ Department of Global Health, University of Washington, Seattle, WA, United States; ^2^ Kaiser Permanente Washington Health Research Institute, Seattle, WA, United States; ^3^ School of Information, University of Michigan, Ann Arbor, MI, United States; ^4^ Brown School, Washington University in St. Louis, Saint Louis, MO, United States; ^5^ Hennepin Healthcare Research Institute, Minneapolis, MN, United States; ^6^ Department of Psychiatry and Behavioral Sciences, University of Washington, Seattle, WA, United States; ^7^ Division of General Medicine and Clinical Epidemiology, University of North Carolina at Chapel Hill, Chapell Hill, NC, United States

**Keywords:** implementation strategies, theorizing, social influence, mechanisms, causal pathway diagrams

## Abstract

**Background:**

Opinion leadership, educational outreach visiting, and innovation championing are commonly used strategies to address barriers to implementing innovations and evidence-based practices in healthcare settings. Despite voluminous research, ambiguities persist in how these strategies work and under what conditions they work well, work poorly, or work at all. The current paper develops middle-range theories to address this gap.

**Methods:**

Conceptual articles, systematic reviews, and empirical studies informed the development of causal pathway diagrams (CPDs). CPDs are visualization tools for depicting and theorizing about the causal process through which strategies operate, including the mechanisms they activate, the barriers they address, and the proximal and distal outcomes they produce. CPDs also clarify the contextual conditions (i.e., preconditions and moderators) that influence whether, and to what extent, the strategy's causal process unfolds successfully. Expert panels of implementation scientists and health professionals rated the plausibility of these preliminary CPDs and offered comments and suggestions on them.

**Findings:**

Theoretically, opinion leadership addresses potential adopters' uncertainty about likely consequences of innovation use (determinant) by promoting positive attitude formation about the innovation (mechanism), which results in an adoption decision (proximal outcome), which leads to innovation use (intermediate outcome). As this causal process repeats, penetration, or spread of innovation use, occurs (distal outcome). Educational outreach visiting addresses knowledge barriers, attitudinal barriers, and behavioral barriers (determinants) by promoting critical thinking and reflection about evidence and practice (mechanism), which results in behavioral intention (proximal outcome), behavior change (intermediate outcome), and fidelity, or guideline adherence (distal outcome). Innovation championing addresses organizational inertia, indifference, and resistance (determinants) by promoting buy-in to the vision, fostering a positive implementation climate, and increasing collective efficacy (mechanisms), which leads to participation in implementation activities (proximal outcome), initial use of the innovation with increasing skill (intermediate outcome) and, ultimately, greater penetration and fidelity (distal outcomes). Experts found the preliminary CPDs plausible or highly plausible and suggested additional mechanisms, moderators, and preconditions, which were used to amend the initial CPD.

**Discussion:**

The middle-range theories depicted in the CPDs furnish testable propositions for implementation research and offer guidance for selecting, designing, and evaluating these social influence implementation strategies in both research studies and practice settings.

## 1 Introduction

Appealing to and winning over the hearts and minds of potential adopters of innovations and evidence-based interventions is often key to successful implementation. Opinion leadership, educational outreach visiting (also called academic detailing), and innovation championing are three common strategies that employ various forms of social influence to support organizational and professional behavior change in health care delivery. Although authors have delineated similarities and differences in the roles, attributes, and skills of the people enacting these strategies (1–4), ambiguities persist in what exactly they do, which implementation challenges they distinctly address, how their actions address these challenges, and how contextual factors influence their effectiveness. These ambiguities stem in part from the frequent conflation of these strategies in empirical studies (2–4) and the often-incomplete descriptions of these strategies in published reports (5). Other contributing factors include limited theoretical grounding, investigator theorizing, and empirical study about how these strategies work and under what conditions they work well, work poorly, or work at all. Resolving these ambiguities could improve the selection, design, delivery, and evaluation of these implementation strategies in both research studies and practice settings.

In this paper, we develop middle-range theories that offer plausible explanations of how these three implementation strategies address organizational and provider-level barriers to the adoption and implementation of innovations and evidence-based interventions. The theories also identify contextual factors that plausibly moderate the effectiveness of these strategies or plausibly serve as necessary conditions for strategy operation. Drawing on key conceptual articles, systematic reviews, empirical studies, and practice-oriented publications for the strategies, we used causal pathway diagrams to theorize about how and under what conditions these strategies produce desired implementation outcomes (6, 7). The middle-range theories depicted in the causal pathway diagrams were rated for plausibility by panels of implementation scientists and health or mental health professionals with expertise in the strategies and were revised based on the feedback they provided. The resulting theories provide a set of testable propositions for implementation research and provisional guidance for deploying these strategies in implementation initiatives.

## 2 Methods

The work presented below was conducted as part of research study funded by the National Cancer Institute (R01CA262325) to identify plausible strategy-mechanism linkages, develop causal models for mechanism evaluation, produce measures needed to evaluate such linkages, and make these models, methods, and measures available in a user-friendly website (8). The research team (i.e., authors), comprised of experts in implementation science and agile science, sought to develop plausible theories of how and under what conditions 30 commonly used implementation strategies work. The Expert Recommendations for Implementation Change compilation served as the foundation for strategy selection (9). We call these theories “middle-range theories” because, unlike “grand theories” such as social constructionist theory or symbolic interactionism, they are theories to guide empirical inquiry and explain specific phenomena, namely, specific implementation strategies.

**Figure 1:**
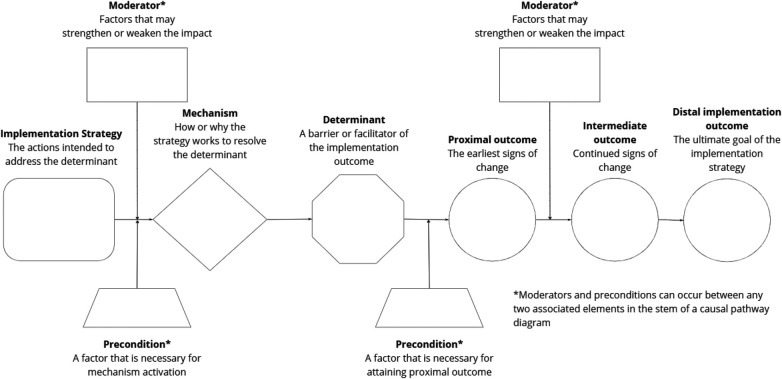
Causal pathway diagram (CPD) template example. The CPD template can be modified to include more elements or fewer elements to reflect current research or thinking about how, and under what conditions, an implementaion strategy works.

The research team used causal pathway diagrams (CPDs) to theorize how, when, and under what conditions opinion leadership, educational outreach visiting, and innovation championing lead to desired implementation outcomes. Like statistical path models and directed acyclic graphs, CPDs are visualization tools to explicitly depict and support theorizing about factors implicated in an implementation strategy's causal process (6). As detailed in Figure 1, a simple CPD has two parts (see Figure 1): (1) a “stem” that describes the main causal process through which a strategy operates, including its mechanism(s) of action, determinants it is intended to address, and the series of outcomes that should be expected if the strategy is operating as intended; and (2) “leaves” that represent contextual factors, specifically preconditions and moderators, that operate as “effect modifiers” to influence whether, and to what extent, the process represented by the stem unfolds successfully (7). The elements that comprise a CPD are defined in Table 1. Constructing a CPD involves five steps: (1) operationalizing the strategy in terms of its core components, particularly those activities that distinguish the strategy from other strategies; (2) identifying the outcomes; (3) identifying the determinant(s); (4) articulating the mechanisms through which the strategy operates on the determinant(s); and (5) articulating effect modifiers (7). We followed these steps.

**Table 1:** Definitions of causal pathway diagram elements (7).

Term	Definition
Implementation strategy	Methods used to improve the adoption, fidelity, or sustained use of an evidence-based treatment, practice, or service.
Mechanism	The process through which an implementation strategy operates to affect an implementation outcome.
Determinant	A factor that can positively or negatively affect an implementation outcome. Determinants are often referred to as barriers or facilitators of implementation. Although implementation strategies could target determinants that operate, or could operate, as facilitators of implementation, more often they target determinants that operate, or could operate, as barriers of implementation. In CPDs, therefore, determinants reflect implementation barrier
Proximal outcome	The most immediate, observable outcome of an implementation strategy.
Intermediate outcome	Intermediate signals that the strategy is continuing to work as intended.
Distal implementation outcome	The downstream implementation outcome that the implementation strategy is ultimately intended to achieve.
Precondition	A factor that is necessary for an implementation strategy to exert its influence on an implementation outcome.
Moderator	A factor that can strengthen or weaken the influence of an implementation strategy.

To develop the CPDs, the research team conducted a structured Deep Dive (10), a common practice in business wherein a team conducts an intensive investigation of a problem, situation, or idea and engages in collective brainstorming and problem-solving. Research team members met in person for 2.5 days in 2023 to present, discuss, and revise the CPDs that they drafted for the first nine strategies (out of a planned 30) listed in the Expert Recommendations for Implementing Change (ERIC) compilation (9), including the three reported here. To prepare for the Deep Dive meeting, each research team member conducted a non-systematic search of the literature and reviewed identified conceptual articles, systematic reviews, empirical studies, and practice-oriented publications for the strategy they were assigned, looking for statements, evidence, or clues about:

• The purpose of the strategy, useful for identifying the distal outcome that could be attained.
• The rationale for the strategy, useful for identifying the determinant (barrier) the strategy could be deployed to overcome.
• The contextual factors that influence the strategy's effectiveness, useful for identifying the preconditions and moderators that influence whether, or how well, the strategy works.
• The strategy's mechanism(s) and proximal outcomes, often the least discussed aspects of strategies.

In addition, research team members noted variations in how the strategy was defined and operationalized, common strategy activities that might constitute core components, and any theories mentioned in relation to the strategy. Team members documented these gleanings from the literature, with citations to sources, in a templated whiteboard in Miro (11), an online collaborative workspace. For each of the three implementation strategies, they then constructed a CPD in Miro, using prompts to theorize about CPD elements not found in the literature (often the case for mechanisms) and theorize the function and location of other elements in the causal process, such as moderators and preconditions.

To provide structure and consistency to the oral presentations, research team members responded in writing prior to the Deep Dive meeting to a series of questions about relevant details they found in the literature, what challenges they experienced constructing the CPD, and how they addressed those challenges (see Supplementary File). They used these responses to contextualize the CPD they presented. After each oral presentation, research team members engaged in 60–80 min of group discussion exploring and refining the CPD. In addition to addressing questions and uncertainties raised in the oral presentation, they sought collectively to:

• Balance the specificity and generality of the descriptions of the strategy, mechanism(s), and barrier(s).
• Consider when and why a strategy might be especially useful compared to other, similar strategies.
• Clarify the causal logic of the proposed mechanism(s) and consider any theoretical support for that logic.
• Differentiate the proposed mechanism(s) from both the barrier(s) the strategy addresses and the strategy's core components to eliminate tautologies.
• Identify the “boundary conditions” or circumstances under which deploying the strategy seems appropriate and feasible.
• Consider the length of the causal chain to ascertain whether the strategy, in isolation, could realistically produce an implementation outcome as a distal outcome.

After the Deep Dive meeting, research team members revised their assigned CPDs using the extensive notes taken by research staff members of the group discussions. These revised CPDs were again presented and discussed in twice-monthly, virtual research team meetings. The CPDs were revised further and prepared for review by subject matter experts.

For the review of the preliminary CPDs generated through the Deep Dive, the research team recruited a panel for each implementation strategy of nine to ten implementation researchers and health or mental health professionals with expertise or experience deploying or evaluating the strategy. Panel size and selection criteria followed guidance for content validity studies for instrument development (12, 13), as we perceived similarities between rating the relevance of draft survey items and rating the plausibility of preliminary CPDs: both tasks involve soliciting evaluative judgments from subject matter experts. The researchers were often the first or senior authors of systematic reviews, conceptual articles, or empirical studies that informed the development of the CPDs. The health or mental health professionals were known by research team members to have practical experience with the implementation strategy. Research team members endeavored to recruit international experts with these qualifications. Team members supplemented this recruitment approach by recruiting additional implementation researchers from the Mechanisms Network of Expertise, a group convened with funding from the Agency for Healthcare Research and Quality to propose a research agenda to advance the study of implementation strategy mechanisms (14). These researchers possessed general expertise in implementation strategies and scientific interest in how strategy work; none had any involvement in the development of the CPDs.

Expert panelists completed a web-based survey in which the strategy's preliminary CPD was displayed and summarized. Using 10-point ordinal scales ranging from “low” to “high”, they rated the plausibility of three elements: the proposed mechanism(s) through which the strategy works, the proposed determinant(s) the strategy addresses, and the proposed outcome(s) the strategy produces. They also rated the plausibility of the entire causal pathway using the same 10-point scale. They could, if they wished, suggest additional mechanisms, barriers, outcomes, preconditions, or moderators, or comment on the plausibility or completeness of the preliminary CPD. Plausibility ratings for CPD elements, and the entire causal pathway, were analyzed by calculating the median and the corresponding interquartile ranges (IQR). Plausibility ratings were interpreted by their median ranking as highly plausible (≥7), plausible (>4–<7), or implausible (≤4) (15). Preliminary CPDs were modified based on the comments and suggestions that experts provided and, at that point, considered “final” for the purposes of this theory development exercise.

## 3 Results

### 3.1 Opinion leadership

#### 3.1.1 Operationalizing opinion leadership

In *Diffusion of Innovations*, Rogers (16) defined opinion leadership as “the degree to which an individual is able to influence other individuals’ attitudes or overt behavior informally, in a desired way with relative frequency”. Researchers have generally embraced this definition verbatim [e.g., (5)] or with minor variations (2, 3, 9, 17). Based on a systematic review of 24 studies, 350 primary care practices, 3,005 health professionals, and 29,167 patients, Flodgren et al. (5) concluded that local opinion leaders alone or in combination with other strategies can be effective in promoting health professional behavior change; however, they noted that the effectiveness of opinion leadership varies both within and between studies. Although these authors could not explain the observed heterogeneity of treatment effects, variation in how opinion leadership was operationalized seems a likely contributing factor (5). As the definitions above indicate, opinion leaders influence peers' attitudes and behavior *informally*; yet opinion leaders in the included studies often engaged in formal activities to educate and influence peers. Formal activities variously included conducting tutorials on the hospital floor, holding formal educational sessions, sending out educational materials, hosting community meetings with recognized experts, participating in community-based task forces, delivering didactic programs, performing outreach activities, formally consulting with colleagues, giving Grand Rounds talks, leading interactive seminar sessions, and signing statements that summarize evidence and provide explicit recommendations. In performing these formal activities, opinion leaders leveraged the attributes that made them opinion leaders, namely their credibility, respect, and position within local networks. However, they were no longer strictly acting as opinion leaders, but rather enacting other implementation strategies, such as educating and advocating. Informal activities that influence others are, by definition, the core components of opinion leadership. The included studies offered minimal detail on the informal activities taken by opinion leaders to promote diffusion of innovation; the activities were variously described as informal consultations, informal group discussions, informal contacts with colleagues, word-of-mouth spread of information, unplanned face-to-face communications, and hallway conversations (5).

#### 3.1.2 Distal implementation outcome and its determinant

Flodgren et al.'s systematic review (5) provides evidence that opinion leadership can improve health professionals' compliance with evidence-based practice. Thus, opinion leadership appears effective for achieving the distal implementation outcome of penetration (18), that is, the spread of evidence-based practice among health professionals. However, neither the systematic review nor other published studies identifies the determinant (barrier) that this implementation strategy targets. Rogers (16) offers a plausible candidate. He notes that potential adopters of an innovation are often uncertain about the likely consequences that will result from their use of the innovation. Although evidence summaries can inform them about the innovation's advantages and disadvantages *in general*, what they really want to know is, “What are the innovation's advantages and disadvantages *in my situation*?” Opinion leaders can address this uncertainty by informally sharing with potential adopters their subjective evaluation of the innovation or evidence-based practice based on their personal use of it (16).

#### 3.1.3 Mechanism

But *how* exactly does such informal sharing of information and advice address the barrier of uncertainty? Rogers (16) suggests a possible mechanism. As opinion leaders share their subjective evaluation of the innovation or evidence-based practice, potential adopters develop their own subjective evaluations regarding its relative advantage, compatibility, and complexity. Rogers (16) describes these subjective evaluations as attitudes that involve both cognition and affect. Thus, a plausible explanation of how opinion leadership “works” is by triggering the formation of a positive attitude toward the innovation or evidence-based practice among potential adopters.

#### 3.1.4 Proximal and intermediate outcomes

To complete the causal pathway from attitude formation to behavior change, we drew upon the Theory of Planned Behavior (19), which posits that behaviors are influenced by intentions, which are determined by three primary factors: attitudes, subjective norms, and perceived behavioral control. Thus, we propose that formation of a positive attitude toward the innovation or evidence-based practice results in a decision to adopt it (like intention in Theory of Planned Behavior) which, in turn, leads to use of it (like behavior in Theory of Planned Behavior). As this causal process repeats amongst the potential adopters in the opinion leaders' interpersonal network, the distal implementation outcome of penetration, or spread of innovation or evidence-based practice use, occurs.

#### 3.1.5 Contextual factors

Theory and research suggest several contextual factors that could influence whether or how well opinion leadership works. Awareness of the innovation or evidence-based practice is an obvious precondition (16). Less obvious, perhaps, is that potential adopters must have some autonomy to make an adoption decision (16). Additionally, opinion leaders earn and maintain their credibility and influence in part through their conformity to local system norms; by exemplifying and expressing system norms, they serve as a role model for others (16). Thus, system norms could influence the extent to which opinion leaders' informal sharing of information and advice triggers the formation of positive, neutral, or negative attitudes toward the innovation or evidence-based practice among potential adopters. The extent to which potential adopters seek information and advice from opinion leaders might also depend on their tolerance of uncertainty (20) or the trialability of the innovation or evidence-based practice (16). The Theory of Planned Behavior (19) suggests that subjective norms and perceived behavioral control could moderate the extent to which a positive attitude toward the innovation or evidence-based practice influences intention (i.e., adoption decision). Rogers (16) suggests that, even with positive attitudes toward the innovation, an action cue might be needed to move from adoption decision (intention) to innovation use (behavior). Action cues can take several forms, including but not limited to clinical reminders. Finally, network characteristics such as size, density, stability, and homophily likely influence the speed and extent to which use of the innovation or evidence-based practice spreads among potential adopters (16, 21). As use spreads, subjective norms may become even more favorable, potentially accelerating the penetration rate.

#### 3.1.6 Expert feedback

**Figure 2:**
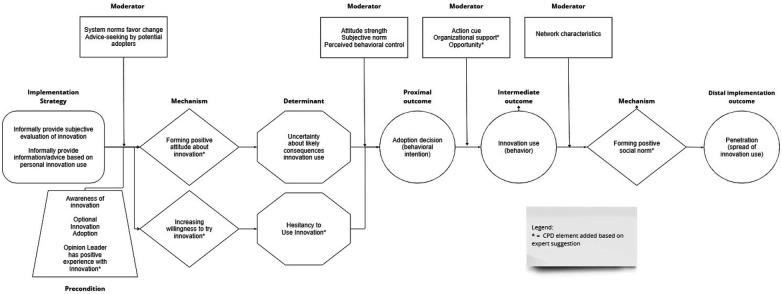
Causal pathway diagram for opinion leadership strategy.

Ten experts rated the plausibility of the various elements of the opinion leadership CPD and the entire causal pathway, commented on the plausibility or completeness of the CPD, and suggested additional mechanisms, barriers, outcomes, preconditions, or moderators (see Figure 2). Four experts had published articles about the strategy; the other six had participated in the implementation science focused Mechanisms Network of Expertise. In terms of demographics, three experts self-identified as male, five as female, and two did not self-identify gender. Seven self-identified as White, one self-identified as Asian, and two did not self-identify race. All ten self-identified as non-Latino or Hispanic.

Using Schmid and Coppieters' interpretive guidance (15), expert ratings indicate the mechanisms, determinant, proximal outcome, intermediate outcome, and distal outcomes depicted in the CPD are either plausible (median <4–<7) or highly plausible (median ≥7), and the entire causal pathway or “stem” of the CPD is plausible (median <4–≥7) (see Table 2). The barrier addressed by opinion leadership exhibited the lowest plausibility rating (median = 6), though still rated as plausible, whereas the mechanism through which opinion leadership “works” had the highest plausibility rating (median = 9).

**Table 2:** Plausibility ratings for opinion leadership causal pathway diagram.

	Mean (SD)	Range	Median	25%-tile	75%-tile
How plausible is it that opinion leadership, when it works, produces the implementation outcome of penetration?	7.00 (1.20)	5–9	7.00	6.25	7.75
How plausible is it that opinion leadership, when it works, addresses the barrier of uncertainty about likely consequences of personal innovation use?	6.88 (1.55)	5–9	6.00	6.00	8.75
How plausible is it that opinion leadership “works” by encouraging potential adopters to form a positive attitude toward the innovation?	8.50 (1.41)	6–10	9.00	7.25	9.75
How plausible is it that opinion leadership, when it works, leads to the proximal outcome of potential adopters making an adoption decision (i.e., form a behavioral intention)?	8.25 (1.28)	6–10	8.50	7.25	9.00
How plausible is it that opinion leadership, when it works, leads to the intermediate outcome of potential adopters engaging in innovation use (behavior)?	7.00 (1.31)	5–9	7.00	6.00	8.00
How plausible do you find the entire causal pathway outlined above?	6.88 (1.46)	5–9	7.00	5.25	8.00

Experts offered 25 comments and suggestions about the CPD and its elements. The main points of feedback centered on the barrier, mechanism, and effect modifiers.

##### 3.1.6.1 Barrier

With respect to the barrier that opinion leadership addresses or plausibly addresses, one expert suggested that the strategy might also address potential adopters' negative attitudes about the innovation. Possibly, but other strategies involving overt, intentional persuasion tactics, such as change advocacy or innovation championing, seem better suited for neutralizing or reversing negative attitudes. In Rogers' (16) view, opinion leaders are more like village elders whose advice and guidance are sought by those uncertain about whether to adopt an innovation or evidence-based practice. The social influence they wield facilitates attitude formation more than attitude change. Another expert suggested the strategy might not address uncertainty *per se* but rather reluctance to face uncertainty. In response, we added a causal pathway wherein potential adopters might still harbor doubts about the likely consequences of personal innovation use, but opinion leaders' social influence and personal example overcomes their lingering hesitancy to use the innovation (barrier) by increasing their willingness or openness to give it a try (mechanism). This pathway might be called the “benefit of the doubt” pathway.

Another expert, citing self-perception theory, argued that behavior determines attitudes, not the other way around. Although there is evidence to support this theory, it does not provide a plausible explanation for how opinion leadership works. However, there could be a feedback loop in which adopters evaluate the consequences of their initial use of the innovation, confirm or disconfirm their initial attitude about it, and either continue or discontinue using it. This would be consistent with Rogers' (16) innovation adoption-decision model.

##### 3.1.6.2 Mechanism

The same expert noted that social norms drive much health professional decision making and behavior and proposed this as the primary way in which opinion leadership works. Drawing on Rogers’ (16) theory and research on opinion leadership, we propose instead that social norms are a downstream mechanism that opinion leadership activates indirectly. When an innovation practice is introduced into a setting, no social norm supporting its use exists. Instead, health professionals uncertain about whether to adopt the innovation seek advice and guidance from opinion leaders, who share their subjective evaluation of it. “I have tried this innovation. Here's my experience with it. Here's what happened when I tried it. Here's my opinion about it”. In a sense, opinion leaders “normalize” (or make normative) innovation use. Over time, however, as more health professionals form a positive attitude and adopt the innovation, a social norm develops. That is, as the innovation diffuses, shared understandings and beliefs develop about the innovation. Thus, opinion leaders could function as “flywheels” for social norm formation and, once formed, those norms could play an important role in driving further diffusion. Therefore, we added social norm formation as a downstream mechanism that explains how opinion leadership produces the distal implementation outcome of penetration.

##### 3.1.6.3 Contextual factors

Finally, experts commented that there are likely additional contextual factors that influence how opinion leadership works well, works poorly, or works at all. In response, we added contextual factors that experts specifically mentioned: namely, the opinion leader has a positive experience with the innovation as a precondition for mechanism activation, and organizational support and opportunity as moderators of potential adopters' capability to act on their intention to use the innovation or evidence-based practice.

### 3.2 Educational outreach visiting

#### 3.2.1 Operationalizing educational outreach visiting

The Cochrane Effective Practice Group defines educational outreach visiting as “use of a trained person from outside the practice setting who meets with health professionals in their practice settings to provide information [about an innovation or evidence-based practice] with the intent of changing their performance” (22). Researchers have generally embraced this definition verbatim [e.g., (23–28)]. Based on a systematic review of 69 studies involving more than 15,000 health professionals, O'Brien et al. (22) concluded that educational outreach visiting alone or in combination with other strategies can be effective in promoting health professional behavior change. Effects on prescribing behavior, the most frequently targeted health professional practice, were consistently small; effects on other health professional practices, such as diabetes management and tobacco counseling, ranged from small to moderate. Other recently conducted systematic reviews that included educational outreach visiting have reached similar conclusions (29–31).

In contrast to opinion leadership, educational outreach visiting is a well-specified strategy. Informed by social marketing, Soumerai (32) outlined key principles for educational outreach visiting which, translated into concrete activities (33), include:

• Investigating baseline knowledge and motivations for current activity
• Focusing programs on specific categories of physicians (e.g., high-volume prescribers)
• Defining clear educational and behavioral objectives
• Establishing credibility through a respected organizational identity, referencing authoritative and unbiased sources of information, and presenting both sides of controversial issues
• Stimulating active participation by physicians in educational interactions
• Using concise graphic educational materials that highlight and repeat essential messages
• Providing positive reinforcement of improved practices in follow-up visits

Typical educational outreach visits involve specially trained physicians or pharmacists holding brief (<15 min), one-time, one-on-one sessions with targeted health professionals at convenient times in their practice settings (28).

#### 3.2.2 Distal implementation outcome and its determinant

Multiple systematic reviews indicate educational outreach visiting can improve health professionals' compliance with evidence-based practice (22, 29–31). Unlike opinion leadership, educational outreach visiting focuses less on encouraging health professionals to try something new and more on encouraging them to either do more or do less of something that they already do (e.g., decrease inappropriate prescribing, increase appropriate prescribing, or both). Moreover, through one-on-one sessions with selected categories of health professionals (e.g., high-volume prescribers), educational outreach visiting focuses on changing individual health professionals’ practice rather than on spreading innovation or evidence-based practice use among health professionals in a network. Thus, educational outreach visiting seems well-suited for, and effective in, achieving the distal implementation outcome of fidelity (18), or adherence to clinical guidelines or evidence-based practice.

Although the goal of educational outreach visiting is often well described in published studies (e.g., decreasing inappropriate prescribing), the determinant or barrier that the strategy addresses is rarely identified explicitly. Invoking Cabana et al.'s (34) conceptual framework categorizing barriers to physician adoption of clinical practice guidelines, Krakower et al. (35) proposes that educational outreach visiting addresses health professionals' knowledge barriers (by providing evidence-based information), attitudinal barriers (by using social influence tactics in communication), and behavioral barriers (by providing actionable recommendations).

#### 3.2.3 Mechanism, proximal outcome, and intermediate outcome

Luetsch et al.'s (26) realist synthesis of educational outreach visiting articulated 27 context-mechanism-outcome configurations that explain how various aspects of educational outreach visiting contribute to the strategy's effectiveness. Yet, the program theory that they developed to integrate the 27 configurations does not illuminate how the strategy “works” to address the abovementioned determinants (barriers) or realize the desired outcome. By transposing the configurations into a CPD, however, the main causal process through which the strategy plausibly operates becomes evident. Specifically, interactive discussion about the targeted health professional practice (e.g., prescribing a particular drug), including dialogue about uncertainties or controversies about the evidence supporting the practice, encourages the health professional to engage in critical thinking and reflection (mechanism) about the practice and their use of it. As Luetsch et al. (26) observe, critical thinking and reflection are critical precursors to intention to change (proximal outcome) and behavior change (intermediate outcome).

#### 3.2.4 Contextual factors

Transposing the configurations into a CPD also highlights contextual factors that could influence the strategy's effectiveness and strategy components that, in turn, influence those contextual factors:

• Health professional participation in the outreach visit (precondition) could be facilitated by visiting the health professional in their practice setting at a convenient time and keeping the visit brief.
• Health professional engagement (moderator) could be influenced by making the educational session content relevant which, in turn, could be facilitated by investigating the clinician's baseline knowledge, attitudes, and behavioral barriers and using that knowledge to tailor the content to the health professional's needs and circumstances.
• Health professional acceptance of the information and recommendations presented (moderator) could be enhanced when a respected, well-trained visitor builds rapport, presents evidence-based information in an unbiased way, acknowledges uncertainties and controversies, and uses well-designed educational materials such as infographic cards and clinic posters.
• Perceived usefulness of recommendation (moderator) could be increased when recommendations are clearly stated, actionable, and tailored to the health professional's needs and circumstances.
• Health professional self-efficacy (moderator) could be enhanced when actionable, tailored recommendations address clinician-identified behavioral barriers.
• Action cues to engage in behavior change (moderator) could be reinforced when visitors repeat key messages and follow up with visited health professional.

#### 3.2.5 Expert feedback

**Figure 3:**
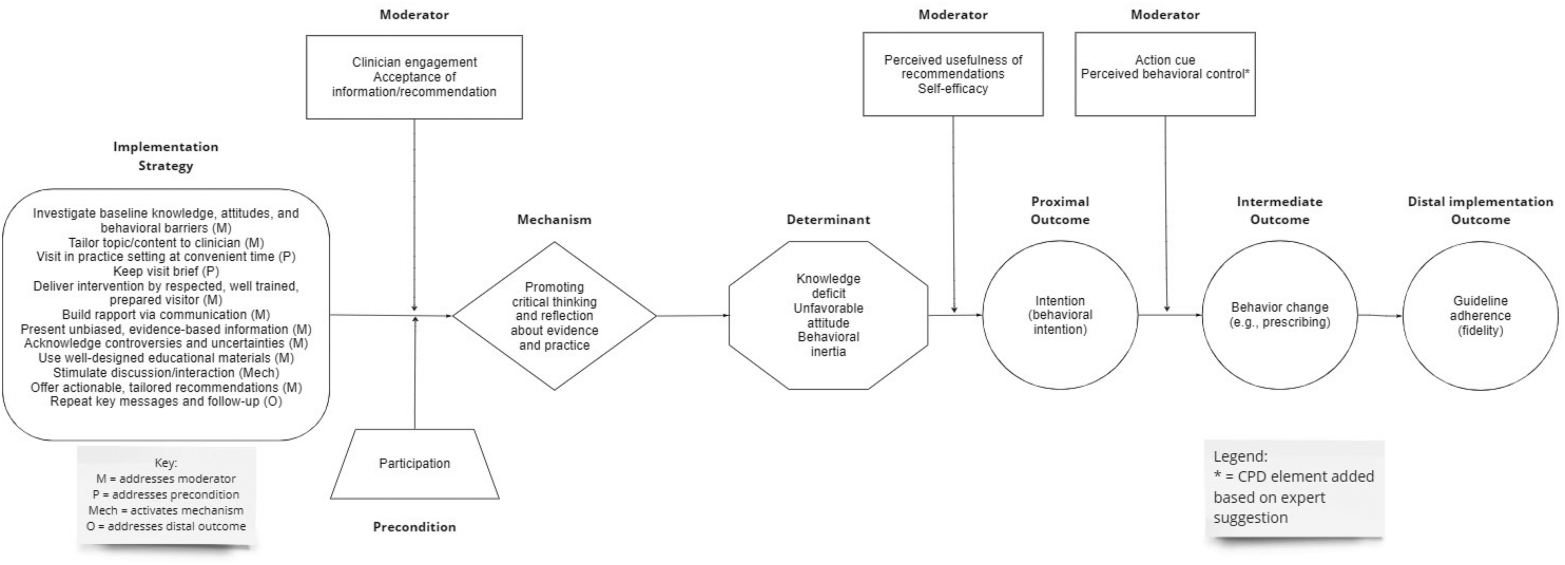
Causal pathway diagram for educational outreach visiting strategy.

Ten experts rated the plausibility of the various elements of the educational outreach visiting CPD and the entire causal pathway, commented on the plausibility or completeness of the CPD, and suggested additional mechanisms, barriers, outcomes, preconditions, or moderators (see Figure 3). Five experts had published articles about the strategy; the other five had participated in the implementation science focused Mechanisms Network of Expertise. In terms of demographics, seven experts self-identified as male, two as female, and one did not self-identify gender. Seven self-identified as White, one self-identified as Asian, and two did not self-identify race. Nine self-identified as non-Latino or Hispanic and one self-identified as Latino or Hispanic. Four of the ten experts also rated the plausibility of the opinion leadership CPD.

Using Schmid and Coppieters' interpretive guidance (15), expert ratings indicate the mechanism, determinant, proximal outcome, intermediate outcome, and distal outcome depicted in the CPD are highly plausible (median ≥7), as is the entire causal pathway or “stem” of the CPD (median ≥7) (see Table 3).

**Table 3:** Plausibility ratings for educational outreach visiting causal pathway diagram.

	Mean (SD)	Range	Median	25%-tile	75%-tile
How plausible is it that educational outreach visiting when it works produces the implementation outcome of fidelity?	8.0 (1.76)	5–10	8.00	6.75	10.0
How plausible is it that educational outreach visiting when it works addresses knowledge barriers attitudinal barriers and behavioral barriers?	7.70 (1.89)	5–10	8.00	6.75	10.0
How plausible is it that educational outreach visiting “works” by encouraging physicians to engage in critical thinking and reflection about the clinical practice and their use of it?	8.56 (1.01)	7–10	8.00	8.00	9.50
How plausible is it that educational outreach visiting when it works leads to the proximal outcome of targeted physicians forming an intention to change their behavior?	8.44 (1.33)	6–10	9.00	7.50	9.50
How plausible is it that educational outreach visiting when it works leads to the intermediate outcome of targeted physicians engaging in behavior change?	7.22 (1.79)	5–10	8.00	5.50	8.50
How plausible do you find the entire causal pathway outlined above?	8.13 ± 1.25	6–10	8.00	7.25	9.00

Experts offered 37 comments and suggestions, primarily focused on contextual factors and mechanisms.

##### 3.2.5.1 Contextual factors

First, experts noted that a host of contextual factors not depicted in the CPD could prevent the health professional from engaging in behavior change, including time availability, competing demands, patient preferences, and organizational constraints, such as staffing. In response, we added perceived behavioral control to the CPD to account parsimoniously for the effects of these and other contextual factors. Perceived behavioral control refers to the perceived ease or difficulty of performing a behavior (19). Consistent with the Theory of Planned Behavior, we propose that contextual factors perceived as barriers or facilitators affect behavior indirectly through perceived behavioral control. We included both self-efficacy and perceived behavioral control in the CPD based on research indicating that (a) these two constructs are conceptually distinct and empirically distinguishable, and (b) these two constructs can have independent effects, with self-efficacy influencing intention and perceived behavioral control influencing behavior (36).

Second, experts suggested that educational outreach visiting is more likely to be effective when (a) the targeted health professional practice is simple, such as prescribing; (b) the evidence supporting it is up to date; and (c) the recommended action based on the evidence is clear. We propose that these factors influence strategy effectiveness through moderators already included in the CPD, specifically: (a) the degree of simplicity of the targeted health professional practice influences health professionals' self-efficacy and perceived behavioral control; (b) the extent to which the evidence is current influences health professionals' acceptance of the recommendation; and (c) the clarity of the recommended course of action influences health professionals' perceived usefulness of the recommendation.

##### 3.2.5.2 Mechanisms

Finally, experts mentioned two scenarios in which health professionals might accept evidence-based information and recommendations without engaging in critical thinking and reflection. In the first scenario, health professionals are convinced of the targeted health professional practice's value due to the strong persuasiveness of the visitor, not due to careful consideration of the pros and cons. In such cases, we argue, the visitor is enacting a different strategy, that of advocate or champion, as use of strong persuasive tactics deviates from the key principles for educational outreach visiting (32, 33). In the second scenario, health professionals accept the visitors' message without thinking critically about it because they have come to trust the visitor (especially after multiple visits) and they do not have strong attitudes or much knowledge about the targeted health professional practice. Although largely conjecture, this notion has implications for potential overlap between the effects of educational outreach visiting and opinion leadership. It is possible that when knowledge barriers, attitudinal barriers, and behavioral barriers are low, the distinction between educational outreach visiting and opinion leadership is not clear cut.

### 3.3 Innovation championing

#### 3.3.1 Operationalizing innovation championing

The Expert Recommendations for Implementing Change strategy compilation describes champions as “individuals who dedicate themselves to supporting, marketing, and driving through an implementation, overcoming resistance that an intervention may provoke in an organization” (9). As early as 1963, management scholar Donald Schon (37) observed that champions are essential for overcoming the organizational indifference, inertia, and resistance that innovations, especially radical ones, encounter when introduced or implemented. In the management literature, innovation champions have been described in heroic terms as individuals who embrace the new idea as their own; promote it with conviction, persistence, and energy through informal networks, and put their reputation on the line to ensure success (37–39). Historically champions were seen as individuals who informally assumed the role of champion for a cause they believed in as opposed to individuals formally appointed by management to play this role. As Shea (4) notes, “in current practice, many organizations appoint individuals to champion roles as an implementation strategy” (40).

Although implementation scientists have embraced the concept and definition of champions elaborated by management scholars, they have focused their attention on clinical champions (40), a role related to, but distinct from, innovation champions. Whereas innovation champions aim to promote organizational *and* professional behavior change by addressing barriers in both the inner setting domain *and* the individual characteristics domain of the Consolidated Framework for Implementation Research (41), clinical champions focus more narrowly on promoting professional behavioral change by addressing individual-level barriers, such as knowledge, skills, attitudes, and perceived norms. To do so, they perform actions other than championing actions, such as educating, training, mentoring, and compliance monitoring. Morena et al. (42) have articulated a theory of how the strategy of clinical champions “works” to address provider-level barriers and promote provider-behavior change. Here we draw upon their work and comparable work by Shea (4) to develop a mid-range theory for the related, but distinct strategy of innovation champions.

**Figure 4:**
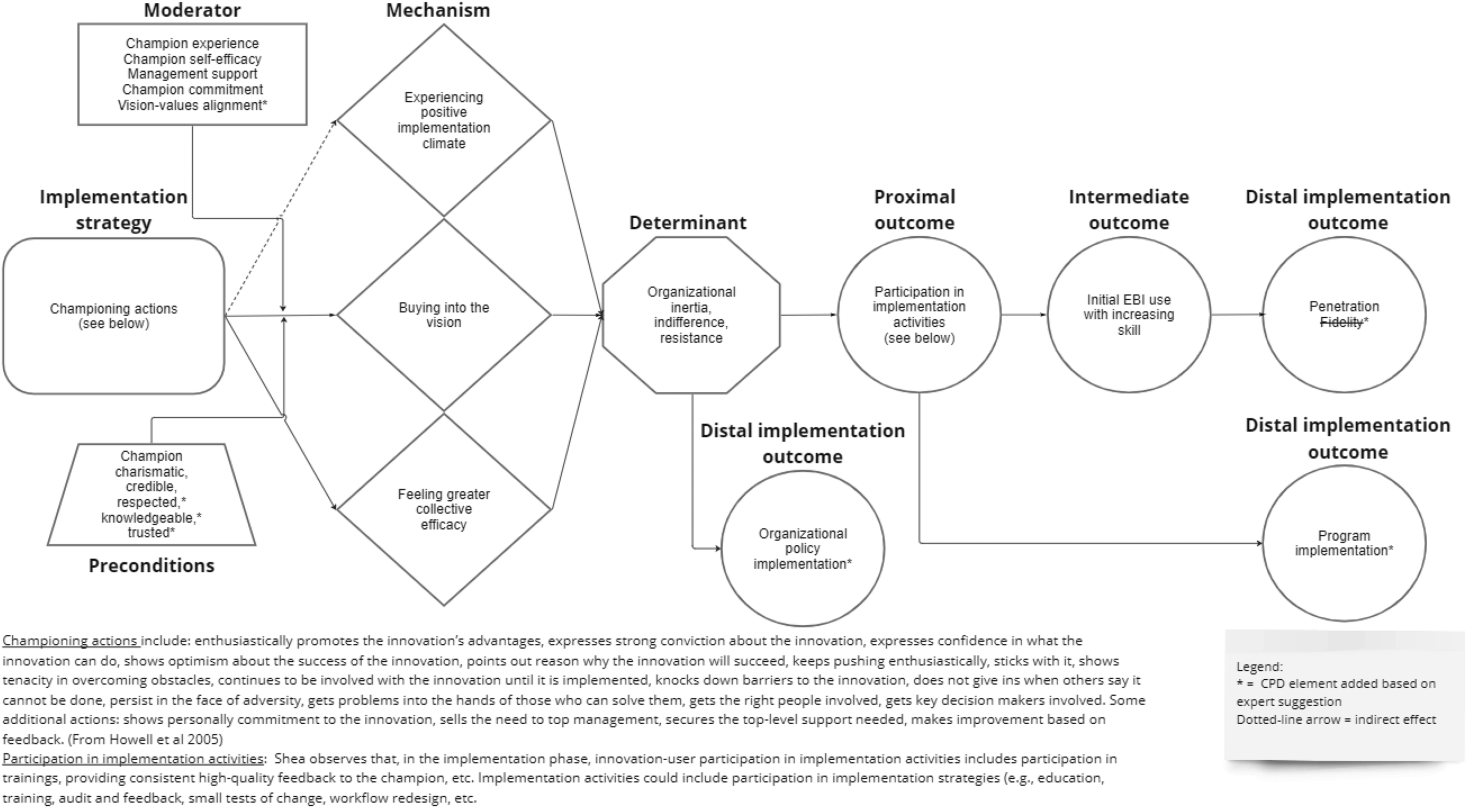
Causal pathway diagram for innovation championing.

At its core, innovation championing involves exerting socio-political influence to get an innovation or evidence-based intervention into practice (38). The strategy can be specified, and differentiated from related strategies, in terms of 14 actions that Howell et al. (43) identified using act-frequency methods and subsequently validated using measure development methods. The actions, listed in Figure 4, fall into three categories: expressing enthusiasm and confidence about the success of the innovation, persisting under adversity, and getting the right people involved. These actions aim to overcome indifference, inertia, and resistance at multiple organizational levels, not just the provider level. Moreover, they reflect both social *and* political efforts to galvanize support for the innovation or evidence-based practice and push the implementation forward.

#### 3.3.2 Implementation outcome and its determinants

Although evidence from primary studies is limited in quantity and quality, systematic reviews conducted by Miech et al. (44) and Santos et al. (45) conclude that champions *can be* effective in both promoting use of evidence-based interventions and increasing adherence to clinical guidelines, which comport with two implementation outcomes: penetration and fidelity (distal outcomes) (18). As already mentioned, as an implementation strategy, innovation championing seeks to overcome organizational indifference, inertia, and resistance to getting an innovation or evidence-based intervention into practice (barriers).

#### 3.3.3 Mechanisms, proximal outcome, and intermediate outcome

How exactly do championing actions address organizational barriers to implementation? Three plausible mechanisms can be derived from the works of Morena et al. (42), Shea (4), and Howell and Higgins (46). These authors propose with varying degrees of emphasis and explicitness that champions shape others' perceptions of the innovation itself, the implementation context, and the implementers' capabilities. For example, champions create and communicate “strategic meaning” around the innovation (i.e., highlight the strategic implications of the innovation for the organization), express confidence in what the innovation can do, and convey optimism that the implementation effort will succeed. In response, organizational leaders and members buy into the champion's vision (mechanism) and embrace innovation implementation. Further, champions get key decision makers involved, sell the innovation to top management, and secure the management support required for implementation. These actions, in turn, generate a positive implementation climate (mechanism), albeit indirectly. Managers respond to the champion's efforts by instituting various implementation policies and practices which, consequently, promotes a shared sense among organizational members, including health professionals, that innovation implementation is expected, supported, and rewarded (47). Finally, champions keep advocating for the innovation enthusiastically, show tenacity in overcoming obstacles, stay involved until the innovation is implemented, and do not give up when others say it cannot be done. These actions instill and reinforce among organizational members, including health professionals, a sense of collective efficacy that they can implement the innovation successfully (mechanism).

Based on Shea's (4) conceptual model of champion impact, an early indicator that the championing strategy is working to overcome organizational indifference, inertia, and resistance is organizational member participation in implementation activities (proximal outcome), such as education, training, task shifting, workflow redesign, small tests of change, and audit and feedback. These activities, over time, result in increasing innovation use with greater consistency and skill (intermediate outcome).

#### 3.3.4 Contextual factors

Studies have identified several characteristics of champions that likely serve either as moderators of strategy effectiveness or as necessary conditions for strategy operation. For example, effective champions are described as personable, charismatic, knowledgeable, respected, trusted, credible, and well-liked (48, 49). Of these various personal qualities, we propose that two plausibly function as preconditions: charisma and credibility. Unlike opinion leadership, championing involves *selling* the innovation (38) or, more accurately, selling the vision of what the innovation could be or do (46). Getting others to buy into their vision, buck the status quo, and follow their lead requires a certain level of interpersonal savvy or magnetism. It also requires a certain level of credibility, which likely depends less on expertise with the innovation or evidence-based practice and more on reputation for success in driving new ideas, technologies, or practices through the process of development, implementation, or both. When champions are formally appointed, their degree of commitment to the innovation and the level of management support they receive likely influence the frequency and effectiveness of their championing actions (4).

#### 3.3.5 Expert feedback

Nine experts rated the plausibility of the various elements of the innovation championing CPD and the entire causal pathway, commented on the plausibility or completeness of the CPD, and suggested additional mechanisms, barriers, outcomes, preconditions, or moderators. Six experts had published articles about the strategy; the other four had participated in the implementation science focused Mechanisms Network of Expertise. In terms of demographics, five experts self-identified as male and four as female. Seven self-identified as White, one self-identified as Asian, and two did not self-identify race. All nine self-identified as non-Latino or Hispanic. One expert also rated the plausibility of the opinion leadership CPD.

Using Schmid and Coppieters' (15) interpretive guidance, expert ratings indicate the mechanisms, determinant, proximal outcome, intermediate outcome, and distal outcomes depicted in the CPD are either plausible (median <4–<7) or highly plausible (median ≥7), and the entire causal pathway or “stem” of the CPD is highly plausible (median ≥7) (see Table 4). Two CPD elements had a lower median plausibility rating than the other CPD elements did: the distal implementation outcome of fidelity (median = 6) and the mechanism of generating a positive implementation climate (median = 7). Based on experts' comments about these two CPD elements, we dropped fidelity as a distal outcome and modified the CPD to visually depict the indirect influence that championing has on implementation climate.

**Table 4:** Plausibility ratings for innovation championing causal pathway diagram.

	Mean (SD)	Range	Median	25%-tile	75%-tile
How plausible is it that innovation championing “works” by gaining buy-in to the champion's vision?	8.22 (2.22)	4–10	9.00	6.50	10.00
How plausible is it that innovation championing “works” by generating a positive implementation climate?	6.89 (2.62)	1–10	7.00	6.00	8.50
How plausible is it that innovation championing “works” by fostering a sense of collective efficacy for innovation implementation?	7.44 (2.46)	3–10	8.00	5.50	10.00
How plausible is it that innovation championing, when it works, leads to the proximal outcome of organizational members participating in implementation activities?	9.00 (1.58)	6–10	10.00	7.50	10.00
How plausible is it that innovation championing, when it works, leads to the intermediate outcome of organizational members using the innovation with greater consistency and skill?	8.33 (1.66)	5–10	8.00	7.50	10.00
How plausible do you find the entire causal pathway outlined above?	7.67 (2.24)	3–10	8.00	6.50	9.50

We made five additional modifications to the distal implementation outcomes, operationalization of the strategy, effect modifiers, proximal outcome, and preconditions in response to the 41 comments and suggestions that experts offered.

##### 3.3.5.1 Distal implementation outcomes

First, we added three distal implementation outcomes that championing can produce. A recently published systematic review indicates that, in addition to increasing innovation use among providers (i.e., penetration), championing can promote the adoption of policies and processes, the implementation of programs and technologies, and the uptake of evidence-based practices by patients following recommendation and training by health professionals per guidelines (45). The latter outcome most closely accords with the implementation outcome of service penetration, a subtype of the outcome of penetration that focuses on the percentage of eligible patients receiving it (18).

##### 3.3.5.2 Operationalization of the strategy

Second, experts commented on the specification of the championing strategy. Specifically, they noted that the amount of work the champion needs to do, and the specific actions they take, likely depends on the characteristics of the innovation or evidence-based practice, the features of the organizational context, and the organizational role of the champion. Depending on the circumstances, the champions might need to engage in goal setting, progress monitoring, progress reporting, and other managerial tasks. Likewise, the champion might need to work hard to create a shared sense of purpose and direction, especially if the champion's vision for the innovation is not well aligned with collective values.

##### 3.3.5.3 Contextual factors

Third, and related to the previous point, we added vision-values alignment as a moderator of mechanism activation based on an expert's comment that the degree to which champion's vision aligns with the organizational members' collective values could influence the extent of buy-in to the vision.

##### 3.3.5.4 Proximal outcome

Fourth, we expanded the list of implementation activities in which organizational members participate (proximal outcome) to include planning, executing, and reflecting and evaluating. Several of these activities figure in the Implementation Process domain of the Consolidated Framework for Implementation Research (41).

##### 3.3.5.5 Preconditions

Finally, based on experts' comments, we added to the CPD three personal qualities of champions—knowledgeable, respected, and trusted—as preconditions for mechanism activation. These three qualities have been identified as characteristics of effective champions (48, 49).

## 4 Discussion

Drawing on published literature and expert input, we developed middle-range theories that offer plausible explanations of how, when, and under what circumstances three common implementation strategies—opinion leadership, educational outreach visiting, and innovation championing—address implementation barriers and produce implementation outcomes. Briefly, we proposed that opinion leadership addresses potential adopters' uncertainty about likely consequences of innovation use (determinant) by promoting positive attitude formation about the innovation (mechanism), which results in an adoption decision (proximal outcome), which leads to innovation use (intermediate outcome). As this causal process repeats, penetration, or spread of innovation use, occurs (distal outcome). Educational outreach visiting addresses knowledge barriers, attitudinal barriers, and behavioral barriers (determinants) by promoting critical thinking and reflection about evidence and practice (mechanism), which results in behavioral intention (proximal outcome), behavior change (intermediate outcome), and fidelity, or guideline adherence (distal outcome). Innovation championing addresses organizational inertia, indifference, and resistance (determinants) by promoting buy-in to the vision, fostering a positive implementation climate, and increasing collective efficacy (mechanisms), which leads to participation in implementation activities (proximal outcome), initial use of the innovation with increasing skill (intermediate outcome) and, ultimately, greater penetration and fidelity (distal outcomes). Experts found the middle-range theories depicted in the CPDs plausible or highly plausible and suggested additional mechanisms, moderators, and preconditions, which were used to amend the CPD.

The middle-range theories developed and discussed in this article offer guidance for selecting, designing, delivering, and evaluating these implementation strategies in both research studies and practice settings. For example, although all three strategies employ various forms of social influence, they address different determinants: uncertainty about likely consequences of innovation use (opinion leadership); knowledge, attitudinal, and behavioral barriers (educational outreach visiting); and organizational indifference, inertia, and resistance (championing). This information can be used to *select* strategies that more precisely target the implementation barriers arising in specific practice settings. Likewise, the identification of plausible mechanism(s) highlights those strategy components or activities that are essential for the strategy to “work”. For opinion leadership, the informal sharing of subjective evaluations is proposed as the “active ingredient”. Accordingly, the strategy's *design* should emphasize informal sharing of information and advice over formal educational activities or formal endorsement. Likewise, identifying the main causal process through which educational outreach visiting “works” clarified which strategy components activate the mechanism and which set the stage for the strategy's success by addressing preconditions and moderators. Contextual factors influencing strategy effectiveness can also be improved by deploying additional implementation strategies. Both opinion leadership and educational outreach visiting, for example, could be more effective if *delivered* as part of a strategy bundle that includes clinical reminders or other strategies that provide an action cue. Finally, monitoring (i.e., e*valuating*) proximal outcomes can provide an early signal of whether the selected strategy, as designed and deployed, is working or whether the strategy needs adjusting to increase mechanism activation or contextual factors influencing strategy effectiveness have been overlooked and need to be addressed.

The articulation in the CPDs of the determinants these strategies plausibly address and the contextual factors that could influence their effectiveness highlights some “boundary conditions” for strategy deployment. By “boundary conditions”, we mean the general circumstances under which deployment of the strategy is appropriate and likely to be successful. Opinion leadership and educational outreach visiting are well suited for simple innovations or evidence-based practices that individual health professionals can adopt and implement on their own with minimal organizational support. Innovation championing, by contrast, is generally better suited for complex innovations or evidence-based practices that organizational leaders must first adopt and that require collective, coordinated behavior change to implement. Moreover, the emergent, informal nature of opinion leadership and, for that matter, innovation championing implies practical limits on the utility of intentionally, prospectively recruiting, appointing, and training organizational members to enact these strategies.

The middle-range theories described here add to the growing body of work in implementation science to clarify how strategies work, for which problems, for which outcomes, and under what circumstances. Some authors have used formal methods to support theorizing, as we do, such as realist evaluation (26, 50) or mechanism mapping (51); others have taken a more informal approach (4, 40, 52). Yet all have started with a specific strategy in mind and used general theories and research evidence to inform their theorizing about determinants, mechanisms, contextual factors, and outcomes. As this strategy-specific theorizing proceeds through the strategies in the ERIC compilation and other inventories, we might learn, for example, that seemingly similar strategies plausibly work through different mechanisms (e.g., clinical championing and innovation championing) or, conversely, seemingly different strategies plausibly work through similar mechanisms (e.g., opinion leadership and clinician peer testimonials). These sorts of nuances might be missed by starting with a general theory like social cognitive theory, for example, to theorize about a group or class of strategies.

The middle-range theories described here have three key limitations that merit discussion. First, like all theories, they offer simpler accounts of the more complex phenomena they seek to explain. Notably absent from the CPDs, for example, are feedback loops and other temporal processes that can give rise to non-linear dynamics or emergent states. More complexity could be added to the CPDs and the theories they depict, but at some cost to their comprehensibility and actionability. Second, the theories presented here are likely incomplete regarding contextual factors, as they only include those moderators and preconditions that were discussed in the literature or nominated by expert panelists. Not included are “universal” preconditions and moderators, such as resource availability, supportive leadership, sufficient time, trusting relationships, and other contextual factors that influence most, if not all, implementation strategies. We omitted them for the sake of parsimony. Researchers and implementers are encouraged to use these middle-range theories as a starting point for developing “micro-theories” that incorporate local contextual factors, including those that operate as “universal” preconditions and moderators, that are likely to influence whether or how well the strategies will work in specific practice settings. Finally, the utility of these theories for research and evaluation is limited in part by the paucity of robust, practical measures, particularly for mechanisms and proximal outcomes. Although our research team and others are working to address this problem, we see potential in the use of qualitative methods to detect mechanism activation and proximal outcomes (53, 54). In addition to measurement limitations, rigorous quantitative assessment of plausible mechanisms of implementation strategies requires sample sizes that are difficult to achieve in the cluster randomized controlled trials typically used to evaluate implementation strategies. However, large-scale “natural experiments” in which, say, a health system implements an evidence-based practice or program at scale could create valuable opportunities to assess mechanisms using quasi-experimental, longitudinal study designs. Moreover, researchers evaluating implementation strategies using cluster randomized controlled trials could still contribute to mechanistic implementation research by employing qualitative or mixed methods to study mechanisms. In closing, the middle-range theories offered should not be viewed as finished products; instead, they could and should be refined further through additional theorizing and empirical testing of the propositions they offer (55). Likewise, they should not be viewed as the final word; theorizing is a creative and inherently subjective (or, in this case, intersubjective) process. Other research teams could generate other, equally plausible middle-range theories of how, when, and under what conditions these implementation strategies work.

## Data Availability

The raw data supporting the conclusions of this article will be made available by the authors, without undue reservation.

## References

[1] Grimshaw JM Eccles MP Greener J Maclennan G Ibbotson T Kahan JP Is the involvement of opinion leaders in the implementation of research findings a feasible strategy? Implement Sci. (2006) 1:3. 10.1186/1748-5908-1-3 16722572 PMC1436013

[2] Cranley LA Cummings GG Profetto-McGrath J Toth F Estabrooks CA. Facilitation roles and characteristics associated with research use by healthcare professionals: a scoping review. BMJ Open. (2017) 7(8):e014384. 10.1136/bmjopen-2016-014384 28801388 PMC5724142

[3] Thompson GN Estabrooks CA Degner LF. Clarifying the concepts in knowledge transfer: a literature review. J Adv Nurs. (2006) 53(6):691–701. 10.1111/j.1365-2648.2006.03775.x 16553677

[4] Shea CM. A conceptual model to guide research on the activities and effects of innovation champions. Implement Res Pract. (2021) 2:1–13. 10.1177/2633489521990443 34541541 PMC8445003

[5] Flodgren G O’Brien MA Parmelli E Grimshaw JM. Local opinion leaders: effects on professional practice and healthcare outcomes. Cochrane Database Syst Rev. (2019) 6(6):CD000125. 10.1002/14651858.CD000125.pub5 31232458 PMC6589938

[6] Lewis CC Klasnja P Powell BJ Lyon AR Tuzzio L Jones S From classification to causality: advancing understanding of mechanisms of change in implementation science. Front Public Health. (2018) 6:136. 10.3389/fpubh.2018.00136 29868544 PMC5949843

[7] Klasnja P Meza RD Pullmann MD Mettert KD Hawkes R Palazzo L Getting cozy with causality: advances to the causal pathway diagramming method to enhance implementation precision. Implement Res Pract. (2024) 5:26334895241248852. 10.1177/26334895241248851 38694167 PMC11062231

[8] Lewis CC Klasnja P Lyon AR Powell BJ Lengnick-Hall R Buchanan G The mechanics of implementation strategies and measures: advancing the study of implementation mechanisms. Implement Sci Commun. (2022) 3(1):114. 10.1186/s43058-022-00358-3 36273224 PMC9588220

[9] Powell BJ Waltz TJ Chinman MJ Damschroder LJ Smith JL Matthieu MM A refined compilation of implementation strategies: results from the expert recommendations for implementing change (ERIC) project. Implement Sci. (2015) 10:21. 10.1186/s13012-015-0209-1 25889199 PMC4328074

[10] Horton-Jones M Marsh E Fumarola S Wright-White H McSherry W Rowson T. Using deep dive methodology to investigate an increased incidence of hospital-acquired avoidable category 2 and 3 pressure ulcers. Healthcare (Basel). (2019) 7(2):59. 10.3390/healthcare7020059 30965660 PMC6627303

[11] Miro. Published online 2023. Available online at: www.miro.com

[12] Grant JS Davis LL. Selection and use of content experts for instrument development. Res Nurs Health. (1997) 20(3):269–74. 10.1002/(sici)1098-240x(199706)20:3<269::aid-nur9>3.0.co;2-g 9179180

[13] Polit DF Beck CT Owen S V. Is the CVI an acceptable indicator of content validity? Appraisal and recommendations. Res Nurs Health. (2007) 30(4):459–67. 10.1002/nur.20199 17654487

[14] Lewis CC Powell BJ Brewer SK Nguyen AM Schriger SH Vejnoska SF Advancing mechanisms of implementation to accelerate sustainable evidence-based practice integration: protocol for generating a research agenda. BMJ Open. (2021) 11(10):e053474. 10.1136/bmjopen-2021-053474 34663668 PMC8524292

[15] Schmid AB Coppieters MW. The double crush syndrome revisited–a Delphi study to reveal current expert views on mechanisms underlying dual nerve disorders. Man Ther. (2011) 16(6):557–62. 10.1016/j.math.2011.05.005 21646036

[16] Rogers EM. Diffusion of Innovations. 5th ed. New York: Free Press (2003). Available online at: https://books.google.com/books?id=9U1K5LjUOwEC

[17] MacEachern L Cranley L Curran J Keefe J. The role of motivation in the diffusion of innovations in Canada’s long-term care sector: a qualitative study. Implement Sci Commun. (2020) 1:79. 10.1186/s43058-020-00069-7 32984845 PMC7513489

[18] Proctor E Silmere H Raghavan R Hovmand P Aarons G Bunger A Outcomes for implementation research: conceptual distinctions, measurement challenges, and research agenda. Adm Policy Ment Health. (2011) 38(2):65–76. 10.1007/s10488-010-0319-7 20957426 PMC3068522

[19] Ajzen I. The theory of planned behavior. Organ Behav Hum Decis Process. (1991) 50(2):179–211. 10.1016/0749-5978(91)90020-T

[20] Roets A Van Hiel A. Item selection and validation of a brief, 15-item version of the need for closure scale. Pers Individ Dif. (2011) 50(1):90–4. 10.1016/J.PAID.2010.09.004

[21] Mittman BS Tonesk X Jacobson PD. Implementing clinical practice guidelines: social influence strategies and practitioner behavior change. QRB Qual Rev Bull. (1992) 18(12):413–22. 10.1016/s0097-5990(16)30567-x 1287523

[22] O'Brien MA Rogers S Jamtvedt G Oxman AD Odgaard-Jensen J Kristoffersen DT Educational outreach visits: effects on professional practice and health care outcomes. Cochrane Database Syst Rev. (2007) 2007(4):CD000409. 10.1002/14651858.CD000409.pub2 17943742 PMC7032679

[23] Van Hoof TJ Harrison LG Miller NE Pappas MS Fischer MA. Characteristics of academic detailing: results of a literature review. Am Health Drug Benefits. (2015) 8(8):414–22. 26702333 PMC4684632

[24] Kunstler BE Lennox A Bragge P. Changing prescribing behaviours with educational outreach: an overview of evidence and practice. BMC Med Educ. (2019) 19(1):311. 10.1186/s12909-019-1735-3 31412928 PMC6693161

[25] Liu S Gnjidic D Nguyen J Penm J. Effectiveness of interventions on the appropriate use of opioids for noncancer pain among hospital inpatients: a systematic review. Br J Clin Pharmacol. (2020) 86(2):210–43. 10.1111/bcp.14203 31863503 PMC7015758

[26] Luetsch K Wong G Rowett D. A realist synthesis of educational outreach visiting and integrated academic detailing to influence prescribing in ambulatory care: why relationships and dialogue matter. BMJ Qual Saf. (2023) 33(1):43–54. 10.1136/bmjqs-2022-015498 37142414 PMC10804006

[27] Yeh JS Van Hoof TJ Fischer MA. Key features of academic detailing: development of an expert consensus using the Delphi method. Am Health Drug Benefits. (2016) 9(1):42–50. 27066195 PMC4822978

[28] Kulbokas V Hanson KA Smart MH Mandava MR Lee TA Pickard AS. Academic detailing interventions for opioid-related outcomes: a scoping review. Drugs Context. (2021) 10:2021-7-7. 10.7573/dic.2021-7-7 34970320 PMC8687092

[29] Chhina HK Bhole VM Goldsmith C Hall W Kaczorowski J Lacaille D. Effectiveness of academic detailing to optimize medication prescribing behaviour of family physicians. J Pharm Pharm Sci. (2013) 16(4):511–29. 10.18433/j3kk6c 24210060

[30] Kamarudin G Penm J Chaar B Moles R. Educational interventions to improve prescribing competency: a systematic review. BMJ Open. (2013) 3(8):e003291. 10.1136/bmjopen-2013-003291 23996821 PMC3758972

[31] Baker R Camosso-Stefinovic J Gillies C Shaw EJ Cheater F Flottorp S Tailored interventions to address determinants of practice. Cochrane Database Syst Rev. (2015) 2015(4):CD005470. 10.1002/14651858.CD005470.pub3 25923419 PMC7271646

[32] Soumerai SB Avorn J. Principles of educational outreach (“academic detailing”) to improve clinical decision making. JAMA. (1990) 263(4):549–56. 10.1001/jama.1990.03440040088034 2104640

[33] Freemantle N Nazareth I Eccles M Wood J Haines A. A randomised controlled trial of the effect of educational outreach by community pharmacists on prescribing in UK general practice. Br J Gen Pract. (2002) 52(477):290–5. 11942445 PMC1314269

[34] Cabana MD Rand CS Powe NR Wu AW Wilson MH Abboud PA Why don’t physicians follow clinical practice guidelines? A framework for improvement. JAMA. (1999) 282(15):1458–65. 10.1001/jama.282.15.1458 10535437

[35] Krakower DS Naja-Riese GM Edelstein ZR Gandhi AD Wahnich A Fischer MA. Academic detailing to increase prescribing of HIV Pre-exposure prophylaxis. Am J Prev Med. (2021) 61(5 Suppl 1):S87–97. 10.1016/j.amepre.2021.05.030 34686295

[36] Ajzen I. Perceived behavioral control, self-efficacy, locus of control, and the theory of planned behavior. J Appl Soc Psychol. (2002) 32(4):665–83. 10.1111/j.1559-1816.2002.tb00236.x

[37] Schon DA. Champions for radical new inventions. Harv Bus Rev. (1963) 41:77–86. Available online at: https://cir.nii.ac.jp/crid/1573105975429416320.bib?lang=en (accessed May 15, 2024)

[38] Markham SK Aiman-Smith L. Product champions: truths, myths and management. Res Technol Manag. (2001) 44(3):44–50. 10.1080/08956308.2001.11671429

[39] Maidique MA. Entrepreneurs, champions, and technological innovation. Sloan Manage Rev. (1986) 21(2):59.

[40] Wood K Giannopoulos V Louie E Baillie A Uribe G Lee KS The role of clinical champions in facilitating the use of evidence-based practice in drug and alcohol and mental health settings: a systematic review. Implement Res Pract. (2020) 1:2633489520959072. 10.1177/2633489520959072 37089122 PMC9924254

[41] Damschroder LJ Reardon CM Widerquist MAO Lowery J. The updated consolidated framework for implementation research based on user feedback. Implement Sci. (2022) 17(1):75. 10.1186/s13012-022-01245-0 36309746 PMC9617234

[42] Morena AL Gaias LM Larkin C. Understanding the role of clinical champions and their impact on clinician behavior change: the need for causal pathway mechanisms. Front Health Serv. (2022) 2:896885. 10.3389/frhs.2022.896885 36925794 PMC10012807

[43] Howell JM Shea CM Higgins CA. Champions of product innovations: defining, developing, and validating a measure of champion behavior. J Bus Ventur. (2005) 20(5):641–61. 10.1016/j.jbusvent.2004.06.001

[44] Miech EJ Rattray NA Flanagan ME Damschroder L Schmid AA Damush TM. Inside help: an integrative review of champions in healthcare-related implementation. SAGE Open Med. (2018) 6:2050312118773261. 10.1177/2050312118773261 29796266 PMC5960847

[45] Santos WJ Graham ID Lalonde M Demery Varin M Squires JE. The effectiveness of champions in implementing innovations in health care: a systematic review. Implement Sci Commun. (2022) 3(1):80. 10.1186/s43058-022-00315-0 35869516 PMC9308185

[46] Howell JM Higgins CA. Champions of technological innovation. Adm Sci Q. (1990) 35:317. 10.2307/2393393

[47] Klein KJ Sorra JS. The challenge of innovation implementation. Acad Manag Rev. (1996) 21(4):1055–80. 10.2307/259164

[48] Flanagan ME Plue L Miller KK Schmid AA Myers L Graham G A qualitative study of clinical champions in context: clinical champions across three levels of acute care. SAGE Open Med. (2018) 6:2050312118792426. 10.1177/2050312118792426 30083320 PMC6075611

[49] Bonawitz K Wetmore M Heisler M Dalton VK Damschroder LJ Forman J Champions in context: which attributes matter for change efforts in healthcare? Implement Sci. (2020) 15(1):62. 10.1186/s13012-020-01024-9 32762726 PMC7409681

[50] Sarkies MN Francis-Auton E Long JC Pomare C Hardwick R Braithwaite J. Making implementation science more real. BMC Med Res Methodol. (2022) 22(1):178. 10.1186/s12874-022-01661-2 35752754 PMC9233332

[51] Kilbourne AM Geng E Eshun-Wilson I Sweeney S Shelley D Cohen DJ How does facilitation in healthcare work? Using mechanism mapping to illuminate the black box of a meta-implementation strategy. Implement Sci Commun. (2023) 4(1):53. 10.1186/s43058-023-00435-1 37194084 PMC10190070

[52] Metz A Jensen T Farley A Boaz A Bartley L Villodas M. Building trusting relationships to support implementation: a proposed theoretical model. Front Health Serv. (2022) 2. 10.3389/frhs.2022.894599 36925800 PMC10012819

[53] The Qualitative Research in Implementation Science (QualRIS) group. Qualitative Methods in Implementation Science. Bethesda, MD: National Cancer Institute Division of Cancer Control and Population Sciences (2019). Available online at: https://cancercontrol.cancer.gov/sites/default/files/2020-09/nci-dccps-implementationscience-whitepaper.pdf

[54] Gertner AK Franklin J Roth I Cruden GH Haley AD Finley EP A scoping review of the use of ethnographic approaches in implementation research and recommendations for reporting. Implement Res Pract. (2021) 2:2633489521992743. 10.1177/2633489521992743 34056611 PMC8153409

[55] Kislov R Pope C Martin GP Wilson PM. Harnessing the power of theorising in implementation science. Implement Sci. (2019) 14(1):103. 10.1186/s13012-019-0957-4 31823787 PMC6905028

