# Supplementary material for: Changing hearts and minds: theorizing how, when, and under what conditions three social influence implementation strategies work
Source: Front Health Serv. 2024 Sep 5;4:1443955. doi: 10.3389/frhs.2024.1443955 (PMC11410765; doi:10.3389/frhs.2024.1443955)
Supplement: Supplementary file 1 [file Table1.docx]

**Supplemental File: Documentation process for CPD leads**

We’ve presented a list of questions that we would like CPD leads to address about their general process and as they walk through the CPD. They fall into three categories:

- What did you find in the literature?
- What challenges did you experience?
- How did you address those challenges?

**The pre-work below includes the intended structure for reporting out on your CPD. The content of your response is the start of the collection of papers for the curated CPD.**

1. Did you observe variation in how the strategy is defined? If so, what were some of the key differences among definitions? How did these differences influence your thinking about the strategy?

|  |
| --- |

1. Did any author specify the core components or key activities of the strategy? If not, could you deduce the core components or key activities from the strategy definition(s)? Did authors operationalize the strategy in ways that diverged from the strategy definition or identified core components?

|  |
| --- |

1. Did authors differ with respect to the level at which the strategy works (e.g., individuals vs groups)? If so, which level seems more/most common? Should we develop a CPD for the other level(s)?

|  |
| --- |

1. Did any author mention a theory that provides a rationale for the strategy (e.g., social influence theory)? If not, were you able to identify any theory that might be relevant? How did you do this?

|  |
| --- |

1. Did any author identify the barrier(s) that the strategy purportedly addresses? This could have been described as the goal of the strategy or the problem the strategy seeks to address. If not, how did you infer what the barrier(s) might be?

|  |
| --- |

1. Did any author identify plausible mechanism(s) or offer an explanation/argument/educated guess about how the strategy “works”? If not, how easy or difficult was it to infer a plausible mechanism? How did you go about inferring the mechanism? What challenges did you experience in identifying or describing a plausible mechanism?

|  |
| --- |

1. Did any author identify contextual factors that act as preconditions or moderators? If so, how did you decide whether the contextual factor was a precondition or a moderator? How did you decide where to locate the precondition/moderator along the causal path?

|  |
| --- |

1. Did you add contextual factors as preconditions or moderators to the CPD that were not mentioned by authors? How did you come up with additional factors?

|  |
| --- |

1. Any difficulties identifying the distal or proximal outcome(s)?

|  |
| --- |
